# Zero Trust for NHIs Based on Robust Identity and Access Management for a Resilient IoT Future †

**DOI:** 10.3390/s26082392

**Published:** 2026-04-14

**Authors:** Sthembile Mthethwa, Moses T. Dlamini, Edgar Jembere

**Affiliations:** 1 School of Mathematics, Statistics and Computer Science, University of KwaZulu-Natal, Durban 4000, South Africa; 2 Defence and Security, Council for Scientific and Industrial Research (CSIR), Pretoria 0001, South Africa

**Keywords:** identity and access management, Internet of Things, non-human identities, security, zero trust architecture

## Abstract

The pervasive adoption of Internet of Things (IoT) devices has profoundly reshaped digital connectivity by enabling real-time data exchange and autonomous interactions on a global scale. While this transformation presents substantial operational benefits, it simultaneously introduces significant security challenges, especially in terms of Identity and Access Management (IAM) for non-human entities, such as sensors, devices, machine agents, and service accounts. Historically, traditional perimeter-based security models, which depend on static trust boundaries and implicit trust for internal actors, have been applied to human identities. However, these models prove inadequate for managing non-human identities. This inadequacy has spurred interest in Zero Trust Architecture (ZTA), an advanced security paradigm based on the principle of “never trust, always verify.” This paper examines the application of ZTA in safeguarding IoT ecosystems, with a particular emphasis on managing non-human identities. The study delves into ZTA’s fundamental principles, such as least privilege, micro-segmentation, continuous monitoring, and identity-centric access control, and evaluates their effective implementation in resource-constrained IoT settings. The research identifies critical implementation challenges and considerations for applying identity-based ZTA within IoT contexts. The findings of this paper underscore that ZTA, when meticulously implemented, provides a robust framework for mitigating the cyber risks inherent in IoT ecosystems. Furthermore, the paper delineates prospective research avenues aimed at integrating ZTA into IoT environments. Ultimately, this study contributes to the expanding body of scholarly knowledge by endorsing Zero Trust as a foundational strategy for contemporary IoT security.

## 1. Introduction

This paper is an extended and revised version of work originally presented at the 2025 IEEE International Conference on Smart Internet of Things (SmartIoT 2025) [1]. The Internet of Things (IoT) has fundamentally transformed the way smart devices, systems, applications, and users interact in the digital world built on the Internet Protocol (IP). This is further accelerated by recent technological developments such as cloud computing and high-speed cellular networks (6G) that connect users, systems, and Internet-enabled devices to exchange data at speeds never seen before. The proliferation of connected IoT devices enhances the ability to collect data and make decisions in real-time, emphasizing their vital importance in the digital landscape [2]. Nonetheless, its prevalence and increased connectivity have introduced significant security vulnerabilities, particularly concerning data privacy and the Identity and Access Management (IAM) challenges of Non-Human Identities (NHIs), i.e., IoT devices, sensors, bots, and service accounts. Other challenges include energy consumption, data management and governance, interoperability, connectivity, and affordability. These challenges are usually caused by the inherent resource-constrained nature of such IoT devices (i.e., considering their processing capabilities, memory capacity, and energy demands), which compels them to connect to the Internet with basic lightweight security configurations [3].

Conventional perimeter-based security models, such as IAM models that rely on pre-defined policies, have performed adequately in specific cases throughout the years to prevent cyber threats. However, the ever-expanding dynamic IoT ecosystem has rendered traditional IAM models useless and inadequate [4]. This is because network perimeters are increasingly blurring, with devices and systems constantly communicating autonomously from anywhere in the world and at any time, often with minimal human intervention. Conventional IAM systems rely on static policies and routine authentication, which are inadequate for IoT environments that demand real-time, dynamic, and context-aware trust decisions [4]. Most IAM systems are centralized, leading to considerable scalability challenges as they face difficulties managing the vast number of connected devices that make up the IoT environment.

This failure of traditional models has necessitated that the research community look beyond perimeter-based security architectures that blindly apportion trust and grant unmonitored global access to devices, systems, or users only if they are within a network perimeter. Perimeter-based security architectures were extended through Virtual Private Networks (VPNs) that also blindly assume that authenticated devices, systems, or users are trustworthy and grant global and unmonitored access to resources. This blind trust has allowed threat actors to use compromised devices or systems or user credentials to siphon confidential data unhindered. The rise in Account Take-overs (ATOs) or compromised user credentials has surfaced because of the weaknesses of outward-facing perimeter controls that assume blind trust for all internal devices and systems. This approach completely ignores the fact that some of the internal devices, systems, or user credentials may already be compromised. To alleviate the issues associated with blind trust, de-parameterization, which does not apportion blind trust but assumes that systems or user credentials may have already been breached, has emerged as a recent and fundamental shift in information security. The work of [5] reviews more security flaws in the IoT environment, such as weak IAM and insufficient data encryption, amongst others. All of these have significant consequences for digital infrastructure design, the types of societal risks incurred, the conduct of business, and the evolution of work in digital settings [6].

Traditional network security operates on a perimeter-centric philosophy. It establishes a clear division: a trusted internal network versus an untrusted external network; every perimeter then serves to safeguard the area it encloses [7]. According to Northcutt et al. [8], a network perimeter is a fortified boundary that may integrate a range of security tools, like firewalls, Intrusion Detection Systems (IDSs), border routers, Intrusion Prevention Systems (IPSs), VPN devices, screened subnets, and Demilitarized Zones (DMZs). However, this defense is inherently unidirectional and outward-facing, assumes that the threat is external, and offers no protection from attacks launched within an already compromised network. This has created a need for Zero Trust solutions that “trust none”, “assumes breach” and verify all access requests to prevent threats on both sides (i.e., internal and external) of the perimeter. Therefore, redefining traditional perimeter security is now more vital [4]. The rise in Zero Trust as a modern security model promises to mitigate the shortcomings of conventional security models by providing comprehensive protection against both internal and external adversaries.

The concept of Zero Trust Architecture (ZTA) is built on the foundation of “trusts none but verifies all”, which came in response to this problem and is heavily dependent on robust IAM. ZTA’s core principles assume that no device, user, or system is inherently trustworthy, even if it exists within the network perimeter and has been authenticated. The essence lies in de-parameterization, meaning trust is no longer implicitly granted based on network location. This recognizes the limitations of using singular, fixed defenses across a large network segment [7]. Instead of assuming blind trust, ZTA enforces continuous verification at strategic points of a transaction to weed out ATOs and lock out unauthorized threat actors. Instead of wide and global unmonitored access, it uses least-privilege access controls for each access request before granting controlled and monitored permission to interact with other devices or systems. ZTA introduces a layered security model that ensures robust protection against potential cyber threats on both sides of the perimeter. The key tenets of ZTA, i.e., “never trust but always verify”, least privilege access, micro-segmentation, continuous monitoring, and resilient IAM. These are critical for IoT, especially for managing access requests from NHIs.

This paper explores the application of ZTA, specifically tailored for securing IoT environments. The emphasis is placed on the challenge of managing NHIs such as autonomous IoT devices, machine identities, and software-based agents. Our objective is to explore and understand the challenges associated with securing a wide range of IoT devices and how ZTA might be used to remediate the inherent cyber risk. The focus of the study is on human identity and access management. This is to ensure that access to sensitive resources and interaction within the ecosystem are restricted to only legitimate devices, systems, or users.

This study investigates and explores ways of overcoming significant challenges like the lack of scalability, interoperability, and legacy systems associated with IoT environments. To overcome these challenges, the paper discusses possible solutions and best practices for implementing ZTA based on robust IAM in IoT environments.

The paper is organized as follows: Section 2 presents an overview of traditional perimeter-based models and goes on to introduce new concepts like ZTA, NHIs, and digital identity. Section 3 explores the applicability of ZTA in IAM for securing IoT environments. Section 4 identifies and discusses the challenges and considerations for the implementation thereof. Section 5 presents a practical use case. Section 6 presents a discussion of the findings, and finally, Section 7 offers concluding remarks and proposes directions for future research.

## 2. Background

### 2.1. Traditional Perimeter-Based Models

Advancements in technology have driven the widespread adoption of innovations such as IoT, cloud computing, mobile technologies, etc. While these innovations offer exceptional scalability, flexibility, and efficiency, they also introduce a new spectrum of security vulnerabilities that require more robust security strategies [9]. This is because conventional perimeter security models are inadequate in identifying and addressing modern vulnerabilities. These models operate on a “blind trust” premise that anything within the network is trustworthy, thereby inherently placing trust in internal users and devices. As a result, an already compromised network allows attackers to freely move laterally with minimal resistance, siphoning confidential data without raising any flags, and leaves the entire network exposed.

**Figure 1:**
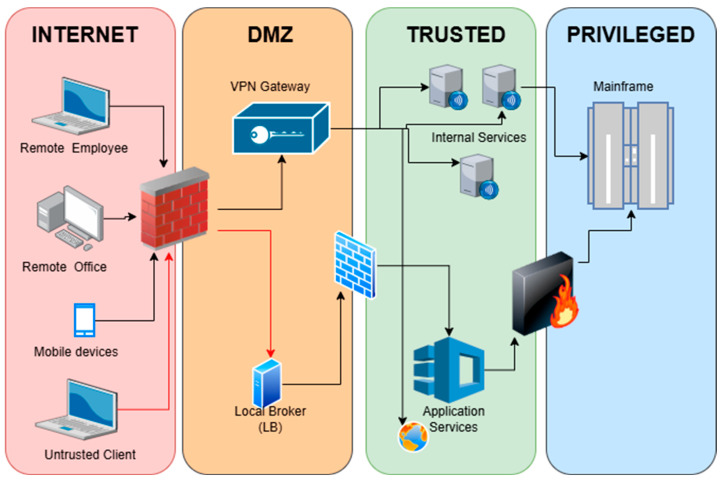
Traditional security architecture [10].

According to [10], traditional perimeter-based security architecture relies on the concept of defense-in-depth, which involves creating several trust zones that are governed by varying rules and levels of access. These zones are typically classified as the Internet, DMZ, trusted zone, and privileged zone, as illustrated in Figure 1. Each zone is protected by perimeter-based controls such as firewalls, VPN gateways, and application-layer security services before access is granted to the mainframe or core systems [10].

The primary challenge with such models is that they are based on the underlying assumption that any entity once authenticated within the network boundary can be trusted. However, this trust is often misplaced, as threats can wait for a compromised user to do the initial authentication and then use an ATO to carry out their malicious activities from the authenticated session. Furthermore, there is a risk of malicious insiders or threat actors who have already bypassed external defenses. Consequently, it becomes essential to move towards a de-parameterized security approach that reduces reliance on network boundaries and instead emphasizes continuous authentication and verification based on identity and context.

The increasing sophistication of cyberattacks targeting enterprise networks has rendered conventional security models inadequate, especially when dealing with threats involving lateral movement and insider attacks [9]. Traditional security models focused on building strong perimeters primarily relied on identity management systems that store credentials such as usernames, passwords, and identity [11]. However, this method does not provide sufficient protection against threats originating from within a compromised network. As a result, it is evident that perimeter-based security paradigms have become ineffective in addressing modern vulnerabilities. This underscores the need to adopt more advanced security models—such as ZTA—that are better suited to combating today’s threat landscape.

Ref. [11] indicates that organizations relying on perimeter-based defenses encountered a 40% surge in breach incidents, in contrast with those that used advanced security strategies such as ZTA. This further highlights the limitations of traditional models and the need for a more dynamic approach. Furthermore, the 2025 report by [12] discusses the dual nature of Artificial Intelligence (AI); while it presents new opportunities, it also introduces additional layers of complexity within an already intricate cybersecurity landscape. Consequently, it has become increasingly challenging for organizations to both embrace and secure AI. Notably, 86% of respondents reported at least one AI-related security incident within the past year, and 48% have recognized the exploitation of AI by malicious actors to enhance their attacks. The same report shows that only 45% of organizations possess the necessary internal resources and expertise to conduct comprehensive AI security awareness, while merely 10% consider AI to be the most challenging aspect of their security infrastructure to protect [12]. As AI-enabled threats continue to evolve in sophistication, the frequency and severity of these threats are expected to escalate. Coupled with the ongoing proliferation of NHIs, this trend is likely to result in an influx of unmanageable threats. Therefore, integrating ZTA into AI-enabled IoT environments can significantly enhance security. The following section provides a detailed analysis of ZTA and its key components.

### 2.2. Zero Trust Architecture

Zero trust architecture has been gaining momentum over the past few years as a security architecture of choice. Ref. [13] distinguishes zero trust and ZTA, where the operative definition of zero trust encompasses the theoretical framework—a set of ideas for rigorously controlling access requests under the premise of an already compromised network. ZTA, conversely, is the practical system architecture built to realize these principles [13]. ZTA is built on the following five basic assumptions [14]:

- The network consistently operates within a hostile environment.
- Threats, both external and internal, are perpetually present on the network.
- The location of the network is not enough to determine its credibility.
- Every device, user, and all network traffic requires rigorous authentication and authorization.
- Security policies should be adaptive and informed by a range of datasets.

A Zero Trust model eliminates inherent trust by emphasizing continuous identity verification, authentication, and authorization [15]. Based on the above assumptions, ZTA is characterized by several basic tenets or core components as depicted in Table 1.

**Figure 2:**
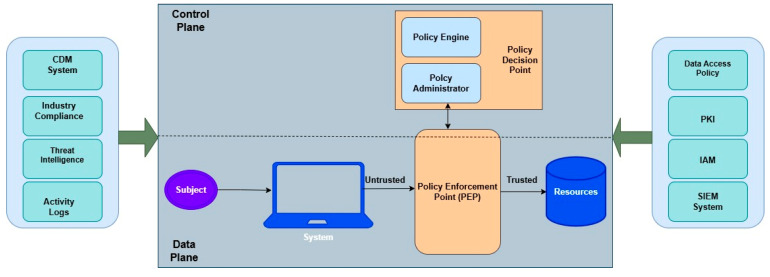
ZTA core components [13].

Figure 2 illustrates the interaction of each of the components described in Table 1. It illustrates a ZTA model that separates the Control Plane and Data Plane to ensure secure access. The Data Plane includes the subject (user or device), the system, and resources, while the Control Plane manages policy decisions through a PDP and enforcement via a PEP. Supporting systems such as CDM, Threat Intelligence, and Compliance, etc., feed contextual data into the model. Access requests start as untrusted and only become trusted after policy evaluation, reflecting the Zero Trust principle of “never trust, always verify”.

A study conducted by [13,16] examined and compiled the fundamental tenets and associated components of ZTA. This identified seven key directions for implementing zero trust:

- Lightweight, scalable approaches for continuous user authentication;
- Granular, context-driven access control measures;
- Data encryption solutions tailored to resource-constrained environments;
- Micro-segmentation strategies to mitigate single points of failure;
- Developing threat-cautious systems combining diverse data and logs;
- Dependable, automated mechanisms for trust evaluation;
- Techniques for enforcing access control at the application level.

In essence, the above list outlines a multi-layered, adaptive, and context-aware approach to cybersecurity, aligning with ZTA principles of continuous verification, least privilege, and resilience against evolving threats. It is evident that identity plays a pivotal role in ZTA, and this is true for both human and non-human identities. This entails providing digital identities and establishing minimal access permissions for subjects. According to [14], it emphasizes security aspects like identity, trust, access control, and permissions, all of which are vital for ZTA. The next section provides a detailed comparison between traditional security models and ZTA models.

### 2.3. Traditional Security Models vs. ZTA Models

Adopting ZTA within IAM presents various advantages and cost implications when compared to traditional security models. Table 2 presents a distinction and attempts to quantify possible gains and costs.

While ZTA approaches might entail higher initial investments and potentially increased operational costs, they provide substantial long-term advantages through enhanced security, scalability, and flexibility. Embracing a ZTA model is especially beneficial, as noted in this comparison, for protecting NHIs in an interconnected world where traditional models may be inadequate, particularly in IoT environments. Zero trust architectures and decentralized identity models effectively tackle IAM challenges in IoT settings by eliminating implicit trust, enhancing continuous verification of device identities, and reducing dependence on centralized credential stores. This enables scalable, tamper-resistant trust mechanisms for machine-to-machine interactions. The decentralization of identity also helps improve the resilience of IAM in that when one identity store has been compromised, subsequent IAM access requests will be channeled to the next available identity store. The next subsection discusses digital identity in the context of NHI with an IoT environment.

### 2.4. Digital Identity

In today’s connected world, digital identity plays a major role in allowing users to access relevant services, make transactions, communicate, etc. This is increasingly true in IoT environments; thus, it is essential to have a robust and reliable digital identity system in place. Digital identity can be defined as the online representation of an individual or an entity, encompassing a range of personal attributes, credentials, and authentication methods used to validate identity across digital platforms [17]. Personal attributes like name, surname, identity number, etc., work for human identities and for NHIs; digital identity attributes can include device type, device name, manufacturer, serial number, etc.

Digital identity systems have evolved over the years from centralized paradigms where identity management systems are managed centrally by a particular organization. This paradigm is prone to a single point of failure, leading to identity theft and becoming exploited by cyber threat actors targeting access to multiple compromised identities. According to [18], the global average cost of a data breach in 2024 reached a record high of USD 4.88 million, representing a 10% increase from the previous year. However, the 2025 report [12] noted a decrease in the global average cost, which fell to USD 4.44 million, marking the first decline in five years. Despite this reduction, the cost remains significantly high, underscoring the importance of investing in secure IAM systems. This investment should prioritize the proper and secure management of digital identities, particularly in IoT environments.

To address some of the shortcomings of centralized identity systems, the emergence of Distributed Ledger Technologies (DLTs) has enabled a decentralized digital identity paradigm that eliminates the dependency on one central entity. DLT-based digital identity distributes credentials to various nodes and helps reduce the risk from cyber threat actors. This also promotes a secure, transparent, immutable, and decentralized digital identity management system [17].

**Figure 3:**
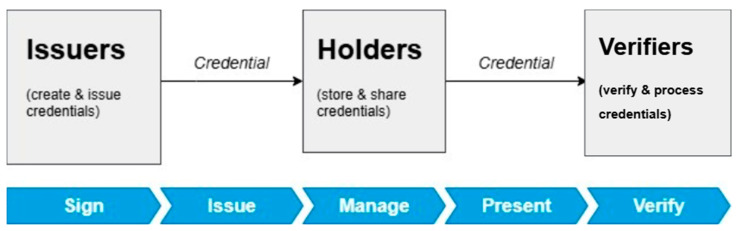
SSI basic roles [19].

Over the years, digital identity owners have had no control over the handling and sharing of their data. Thus, a recent paradigm called Self Sovereign Identity (SSI) was introduced as a solution, which helps reduce privacy concerns related to traditional identity management systems by giving entities, such as natural and legal persons, but also things, such as devices or systems, the authority over their digital identities [19]. In this paradigm, entities have full control of their digital identities and can now choose how their data is shared and with whom. This addresses the issues of data privacy and enhances security. SSI is decentralized and utilizes the concept of Verifiable Credentials (VCs) and Decentralized IDentifiers (DIDs) [19]. VCs allow for the creation of a verifiable claim about an entity, which enables users and devices to authenticate themselves without disclosing unnecessary information [19]. Whilst DIDs enable objects or devices to have their own unique identifiers. This is crucial in IoT environments where there are multiple devices. Furthermore, SSI provides the ability to interact securely and autonomously within a network. This paradigm promotes and is built on the concept of Privacy by Design. This is a principle that promotes the integration of privacy and data protection from the early design stages of systems, technologies, and processes [19]. In the SSI paradigm, there are three roles, namely issuer (creates, signs, and issues credentials or digital identities), holder (owner of VCs or digital identities), and verifier (verifies and processes credentials or digital identities shared by a holder), as depicted in Figure 3.

The use of digital identities in IoT networks is gaining traction because of the built-in benefits that it promises in managing NHIs. It is therefore crucial to adopt the approach in building a secure and robust IAM system to manage NHIs in IoT environments. With its decentralized nature, SSI works well when combined with DLTs, and this can be observed in recent proposed approaches that integrate the two technologies for the identification and management of IoT devices [20,21,22]. The integration of DLTs, SSI, and IoT could unlock new possibilities for the management of NHIs. This integration would provide a seamless and secure way to authenticate identities in real-world scenarios.

Non-human identification is a crucial function within an IoT environment for validation purposes [3]. This validation verifies that every device possesses its own identifiable traits, promoting secure communication and efficient management within the IoT ecosystem [3]. The use of VCs and DIDs assigned to each device enables its recognition, tracking, and verification within the IoT environment. Therefore, proper identification requires a robust identity that is tightly coupled to the device with a unique set of attributes that distinguishes one device from another within the IoT network. Thus, proper identification and authentication of devices play a vital part in creating links or connections between individuals, facilitating the identification of various objects, and assisting administrators in effectively managing these devices. The following are the key properties that should be considered when designing a robust and secure digital identity [3]:

- Unique—a distinct identity that distinguishes itself from other identities in the network. This is paramount for accurate identification while ensuring nonrepudiation, monitoring, auditing, and accountability inside a system.
- Meaningful—the identity should provide relevant details about the owner of the identity. This permits rapid recognition and understanding of the device’s function, which is especially crucial in large-scale IoT installations.
- Concise—identifiers should be brief and straightforward, comprising only the relevant information for identification and management objectives. Thus, minimizing overhead and consumption of resources.
- Secure—identities must be robust and not susceptible to falsification to ensure secure identities and therein help to identify compromised and unauthorized devices, minimizing possible harm to the network.
- Easy to produce—this permits rapid, efficient, and lightweight creation of digital identities, which is vital for ensuring the scalability and seamless operation of digital identity systems, particularly in settings where a high number of identities must be generated and maintained.
- Easy to manage—the capacity to efficiently administer, update, monitor, and decommission identities during their lifetime. This is critical for ensuring that identities can be monitored, maintained, and secured without adding unnecessary complexity or resource expense.

### 2.5. Human vs. Non-Human Identities

Human identities have been significantly studied over the past decade, and this is observed over their evolution. It has progressed beyond basic username and password pairs to elaborate multi-dimensional constructs [23]. This includes the use of biometric traits (fingerprints, facial, voice, etc.), contextual factors (location, device, time of day, etc.), historical activity patterns, behavioral patterns (typing cadence, mouse movements, application usage), and social and professional attributes (role, department, etc.) [23]. As technology continuously evolves, human identities are improved to secure and continuously evaluate trust. Secure digital identities integrate the requirements of privacy and trustworthiness. Privacy in this context ensures that only authorized persons, institutions, or systems can access a digital identity and the information contained therein. Trustworthiness pertains to the accuracy of the digital identity’s information, i.e., that the characteristics accurately represent the entity with a certain level of assurance [24].

While human identities often receive significant attention in security practices, the opposite is observed for NHIs, with organizations not consistently applying the necessary measures to ensure their ongoing security. As digital ecosystems continue to increase rapidly, in 2025, organizations are projected to see growth in cybersecurity challenges due to the increase in NHIs, with zero-day attacks emerging as one of the most critical challenges of security. In the past year, half of the organizations surveyed in [25] reported experiencing security breaches stemming from compromised NHIs. The consequences of these incidents included delays in application launches for 51%, system outages for 44%, and unauthorized access for 43% [25]. According to [23], NHIs are increasing twice as fast as human identities. It is reported therein [23] that enterprises are expected to manage an average of over 250,000 identities by 2025. This is extremely concerning and challenges the ability of organizations to fully manage and secure such a large number of identities. NHIs refer to entities or physical objects that operate independently of direct human interaction, such as Application Programming Interfaces (APIs), IoT devices, service accounts, sensors, and Artificial Intelligence (AI) agents. These are increasing rapidly, so conventional identity management methods are struggling to keep up. Thus, it is crucial to invest in establishing a secure IAM system that caters to the proliferation of NHIs. Unlike human identities in IAM, NHIs operate autonomously, engage in machine-to-machine communication, and use technical identities rather than credentials linked to personal attributes. While humans authenticate using passwords, biometrics, or multi-factor methods with access decisions based on roles, responsibilities, and organizational context, IoT devices authenticate using certificates, cryptographic keys, hardware attestation, or embedded identifiers.

The current state of affairs reflects that authenticating NHIs in IoT has extended the scope of user-based models to include machine or non-human entities. This often requires secure handover between networks and often happens without human intervention [26]. Therefore, today’s IAM for IoT must be interoperable and standardized to work across different networks and edge nodes for them to scale and adequately cater to the needs of NHIs. The work of [27] provides a detailed classification and analysis of current IAM for NHIs. This work classifies current IAMs for NHI and also discusses those that may be necessary in the unfolding future. Ref. [28], coming from the context of smart cities, argues that today’s IAM for securing NHIs in IoT may be accomplished via lightweight token-based authentication, mutual and dynamic authentication protocols (for secure handshake before connection is established), and digital certificates. This work also advocates for ZTA to constantly verify every access request from each IoT device assigned a unique identity, which is often achieved through digital certificates. ZTA goes beyond the norm of establishing and validating the authenticity of NHIs at a point in time. It also captures the behavior, establishes a baseline of typical behavior, and uses it to monitor and flag activities of authenticated IoT devices that deviate from the norm.

With the rise in ZTA, which promotes the importance of NHI governance [29], IAM systems can be improved to cater to these identities and ensure they are secure. This paper aims to investigate the use of ZTA for NHI-based IAM in IoT environments. When implemented correctly, this can improve and secure IoT devices. However, it is crucial to ensure that NHIs are defined correctly, ensuring the uniqueness of features, making it difficult to imitate. The following section explores existing research on implementing ZTA within IAM for enhanced IoT identity security.

## 3. ZTA for Securing IoT Devices

The rise in ZTA has led to an increased interest in its application towards securing IoT devices. According to [30], IAM is a foundational component of ZTA, ensuring authentication and authorization of users and devices according to verified identities and roles. IAM enables systems to dynamically control access by considering factors like user roles, device security posture, and relevant contextual data, thereby protecting sensitive data and critical systems against unauthorized access [30]. The use of ZTA in IoT environments has been introduced in various use cases.

Authors of [30] introduced a tailored ZTA for healthcare systems in smart cities, addressing the security challenges posed by interconnected infrastructures. The model enforces continuous access validation through micro-segmentation, IAM with Multi-Factor Authentication (MFA), and device trust verification, which significantly reduces vulnerabilities. Their experimental results demonstrate a 75% reduction in incident response time and an 11% improvement in detection accuracy [30]. The performance evaluation was conducted using various metrics, including latency, system throughput, incident response, detection accuracy, and resource utilization. While the results suggest significant efficiency, it is important to consider the conditions under which these metrics were measured to determine their applicability to the real world.

In [31], the authors mention the insufficiency of current security controls in addressing the challenges of heterogeneous IoT networks. IoT device data faces numerous threats during transmission. This paper, therefore, reviews security issues inherent in IoT implementation and proposes a framework leveraging Zero Trust and blockchain. It is aimed at assisting IoT implementers resolve current security challenges. However, the effectiveness of the framework in a practical IoT network environment has not yet been evaluated. The authors confirm that it will enhance trust in the future’s interconnected smart environment [31]. In [32], the authors propose a ZTA tailored for IoT systems and develop an access control framework that identifies and manages the various interactions. It represents an initial effort to develop a precisely defined framework; however, its scope focuses solely on access control.

According to [33], the ineffectiveness of conventional perimeter security necessitates novel cybersecurity approaches for IoT. This paper proposes a zero-trust framework incorporating MFA and Single Sign-On (SSO) to build a resilient and flexible security system for IoT environments. The framework was compared with traditional architecture, and the analysis showed its enhanced ability to handle a wide range of IoT security concerns [33]. Therefore, highlighting the relevance of ZTA in securing IoT devices.

In [34], the authors investigate how 5G technology is anticipated to revolutionize the IoT ecosystem by offering extensive connectivity for countless devices coupled with minimal latency and affordable access. The paper explores how 5G, zero-trust, and blockchain are set to propel innovation in Future IoT (FIoT). The proposed architecture aims to enable large-scale protected device authentication [34]. However, there are still challenges that require further research, like scalability, interoperability, deployment, etc.

Another problematic research area is the protection of a multi-cloud environment [35], where conventional perimeter-based security models have also become ineffective. The implementation of ZTA offers an essential solution to address emerging cyber threats even in multi-cloud environments. The paper [32] examines the application of ZTA within multi-cloud environments, emphasizing thorough identity verification, continuous monitoring, and detailed access control. Case studies demonstrate how ZTA implementations can effectively protect multi-cloud environments by minimizing potential points of attack. The authors [32] concluded by analyzing performance trade-offs and outlining best practices for organizations implementing zero-trust security [35].

The use of ZTA for IAM in IoT is observed in [36], which emphasizes how device behavior analysis can boost authentication and authorization processes. However, it recommends further research aimed at examining user and device behavior for dynamic IoT deployment. This approach also integrates factors like changing environmental conditions and malicious activities into ZTA and access control to strengthen defenses against cyber threats.

Another study presented in [37] focuses on the Industrial Internet of Things (IIoT) domain. This study [34] proposes a two-layered ZTA that surpasses conventional perimeter security. It provides an architecture with layered authorization enabling granular access and enhanced scalability. This architecture has two stages: one that manages access to edge services, and the second one governs access to edge resources.

A study conducted by [38] presents a Zero Trust security system that is tailored for smart home IoT environments. This focuses particularly on access control within IAM. The proposed system in [35] ensures that users, devices, and access requests are verified independently of network position or historical behavior. A study by [33] uses affordable components like ESP32 microcontrollers to demonstrate the practical aspect of their solution. The solution proposed therein [33] enforces real-time context-aware access control. This means that before a user, a device, or a sensor could be granted access to a resource, the solution must capture and verify the context of each request over and above the identity. This solution also employs edge-level decision-making for improving privacy, reducing latency, and reducing cloud reliance. This is ideal for resource-constrained IoT devices. However, the system’s reliance on rule-based policies might miss detecting nuanced behavioral anomalies. Rule-based policies are static and cannot detect anything outside the scenarios that are captured in the rules. This is one of the major shortcomings of this work. Furthermore, the insufficient cryptographic measures at the device level indicate potential vulnerability. It must be mentioned, though, that including cryptographic measures might also increase latency. The results of this work also show a lack of scalability and interoperability with larger IoT networks.

In [39], a ZT-IoTrust framework is introduced. This is designed to enhance IoT security through zero-trust principles and quantum-resistant mechanisms. It incorporates a durable lattice-based cryptographic protocol. It also provides a dynamic trust evaluation system for real-time device assessment and an adaptable access control architecture suitable for resource-constrained IoT environments. Experimental evaluation demonstrates a 92.5% cyberattack detection rate, 1.2% false positives, and 0.5% privacy leakage, alongside robust scalability and response times. Though these results are impressive, the framework focuses primarily on access controls and lacks a comprehensive IAM approach. Its reliance on general cyberattack datasets fails to capture protocol-specific behaviors for other communications, which limits its applicability in diverse IoT deployments.

This study [40] examines the integration of ZTA with Machine Learning (ML) to fortify IIoT environments. It effectively addresses industrial constraints by exploring IAM, micro-segmentation, and anomaly detection. An ML-enhanced trust algorithm is proposed to automate access control decisions and to enhance IIoT resilience against cyber threats. The study employs a reinforcement learning-based trust management model, which offers a dynamic alternative to static rule-based frameworks. This capability allows it to effectively respond to real-time cyber threats. While it highlights limitations of traditional perimeter-based security models, it primarily focuses on the policy engine aspect of ZTA, which narrows its scope. Nonetheless, it makes a significant contribution to the development of adaptive and scalable security solutions for IIoT that go beyond static rule-based systems. The proposed solution in [35] systematically reviews cybersecurity challenges and emphasizes zero-trust principles for managing heterogeneous devices and integrating legacy infrastructure.

Similarly to the work of [35,36], another ML-based study [38] proposes a scalable, robust, and reliable novel reinforcement learning approach for securing IoT devices in edge computing. It utilizes an epsilon-greedy search Q-learning method with a proper task scheduling mechanism. This method enhances network performance by addressing noticeable drops in metrics like packet delivery ratio and throughput. The experiments demonstrate a low false positive rate of <2%, which is close to the results of [34]. However, there is a lack of detail on datasets and comparable methods. Further exploration of resource-constrained devices is also required to strengthen the findings of [36].

In [41], the growing adoption of ZTA is discussed as a solution to the limitations of traditional perimeter-centric security architectures. While ZTA has made significant progress in supporting users and general-purpose computing devices, it falls short in effectively managing IoT devices. The paper addresses this gap by introducing an authentication method that constructs unique fingerprints based on IoT device network traffic. These fingerprints reflect device behavior and are used to verify identity by matching against a database, with Software-Defined Networking (SDN) switches regulating network access accordingly. The authors assert that the predictable nature of IoT traffic makes this method suitable for enhancing ZTA’s identity management component. However, evaluation and testing across diverse IoT environments would strengthen the claims and reliability.

In [42], the authors present the SmartIoT Hybrid ML Model, which addresses IoT authentication challenges. This solution integrates Attribute-Based Authentication with lightweight ML techniques for real-time anomaly detection. The results reflect high accuracy and efficiency. Ref. [38] uses Attribute-Based Credentials and Attribute-Based Signatures to preserve privacy. Their [38] experimental results demonstrate high authentication accuracy, precision, and recall, making it suitable for low-powered IoT devices. Although the solution in [38] demonstrates high performance scalability in large IoT networks, it also requires optimization methods to further improve its accuracy.

In [43], the authors propose a ZTA with a novel authentication protocol that uses physical unclonable functions (PUFs) for device verification. This protocol adds continuous location-based access control for secure device access. This is similar to the real-time context-aware access control proposed in [33]. Ref. [39] differs from most of the solutions in that it also employs a three-factor authentication to mitigate credential theft. This effectively guarantees the security of subsequent communications. Experimental analysis therein [39] confirms robustness against known cyberattacks, while performance evaluation indicates low computational costs with better security and low latency. This work [39] presents an ideal case for resource-constrained IoT environments. However, despite its promise, the scheme’s effectiveness in diverse and large IIoT environments warrants further exploration.

Another framework presented by [44], a Zero-Trust Blockchain-Enabled Framework (ZT-BlocIoT), addresses scalability and security in IoT Networks. This framework integrates zero-trust principles with blockchain and AI optimization. It uses dynamic trusted gateways and Deep Reinforcement Learning (DRL)-driven sharding mechanisms to enhance continuous authentication and scalability. Evaluation results confirm superior performance, achieving 2500 transactions per second (TPS) at 700 nodes and reducing cross-shard transactions. However, this also has some limitations. For example, potential targeted adversarial attacks on the DRL agents could manipulate policy updates. Moreover, there are edge–cloud communication delays that might hinder timely trust score synchronization in highly dynamic networks.

Ref. [45] presents a dynamic zero-trust access control model for IIoT. Similarly to the work of [33,39] that adds context-aware access controls, Ref. [42] emphasizes real-time adaptability by integrating device status, network conditions, and user behavior as its context. The solution in [42] effectively manages access in small-scale networks by using a mathematical threat assessment with Fuzzy Logic-Based State Management (FSM). The key features of this solution include a comprehensive context-aware access control mechanism, immediate incident response, and strategic network segmentation. The authors demonstrated that the framework effectively mitigates common cybersecurity threats, such as credential theft, insider threats, compromised IIoT devices, and anomalous user behaviors, without introducing significant latency or computational overhead. However, the framework requires further development to support large-scale IIoT deployments, including hierarchical or distributed control models for PEPs and PEs, distributed policy synchronization, and decentralized anomaly detection.

The reviewed research studies acknowledge and demonstrate the potential viability of incorporating ZTA into IAM for IoT devices. It is no surprise, then, to see a study by [46] highlighting the rise in adoption of ZTA to address IAM challenges for the IoT environment. However, there is still much to be accomplished in this area. It is important to learn from the reviewed research studies. Therefore, this paper learns from existing frameworks and further identifies challenges and considerations for successful future implementation.

## 4. Challenges and Considerations for Implementing ZTA

**Figure 4:**
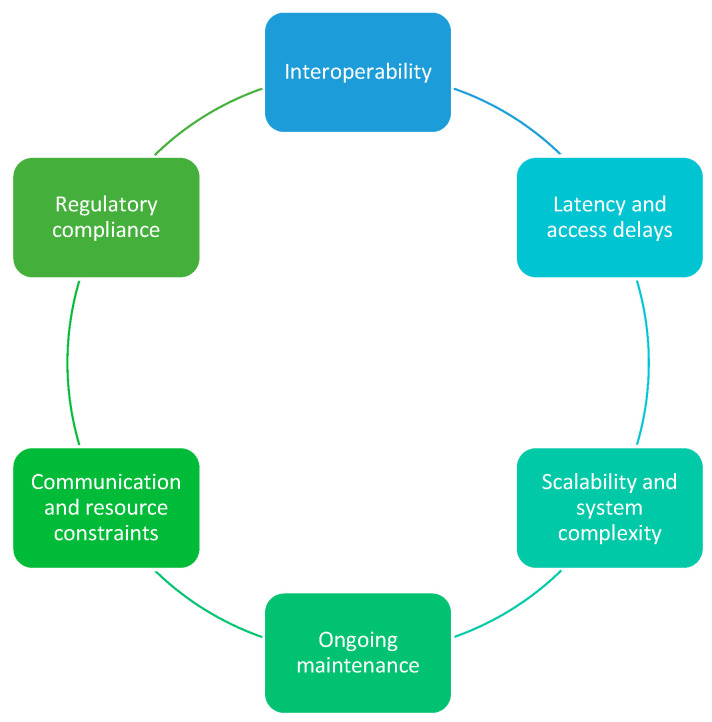
ZTA challenges for ZTA in IoT.

The implementation of ZTA offers numerous advantages over traditional perimeter-based security approaches. These include continuous authentication and verification processes, segmentation of the network into smaller components to deter lateral movement (micro-segmentation), and reduction in the risk of extensive cyberattacks (i.e., blast radius), thereby minimizing the likelihood of data breaches. Additionally, ZTA provides defined access control policies, protections against insider threats, future-proofing of security measures, and continuous monitoring and logging of network traffic. Nevertheless, the successful implementation of ZTA in an IoT environment presents unique challenges that may impede its effective utilization and the realization of its full benefits. These challenges primarily arise from the heterogeneity, inherent scale, and resource constraints of IoT devices. Unlike traditional infrastructures, IoT environments comprise highly diverse devices with varying computational power, connectivity protocols, and security postures, complicating the application of ZTA. Figure 4 illustrates the primary challenges associated with implementing ZTA in IoT environments.

- Interoperability—Device-network incompatibility poses a significant hurdle, particularly concerning legacy systems utilized within operational technology domains. Such technologies often lack contemporary security features, creating challenges for the seamless integration of ZTA. Consequently, it is imperative to facilitate encryption and authenticated communication between these systems and the ZTA network without necessitating alterations to the existing infrastructure. This approach ensures secure interactions between IoT devices and the ZTA environment, thereby providing robust security while preserving operational continuity [30]. Additionally, there exists a deficiency in adequate authentication and authorization models for legacy IoT devices [47].
- Latency and access delays—continuous authentication and micro-segmentation, while critical for enhancing security in IoT ecosystems, can introduce additional processing overhead and network latency. These mechanisms require frequent identity verification and dynamic policy enforcement across segmented network zones, which can slow down data transmission and access requests. In real-time IoT devices and applications, milliseconds of delay can significantly impact system performance, responsiveness, and reliability. This challenge becomes even more pronounced as IoT environments scale, with an ever-increasing number of interconnected devices generating high volumes of data. Any delay in granting access or transmitting information can cascade into postponed decision-making processes, potentially compromising operational efficiency, safety, and service quality [30]. Therefore, balancing robust security measures with low-latency performance is essential for sustaining the functionality of time-sensitive IoT systems.
- Scalability and system complexity—expanding ZTA across multiple facilities presents the challenge of increased system complexity and administrative overhead. Therefore, streamlining this to accommodate the scaling of IoT devices in the future is crucial [30].
- Ongoing maintenance—ZTA requires continuous monitoring and management of security controls, user access, and device compliance. It is important for organizations to invest in resources and establish processes for regular audits and updates. Due to the amount of data that is collected, resources are vital to identify and respond to genuine threats timeously. This is also increased due to the volume of IoT devices on the network. Therefore, using lightweight agents to collect data and send it for analysis, enabling real-time anomaly detection, is crucial.
- Communication and resource constraints—the implementation of ZTA within IoT is significantly hindered by constraints related to communication protocols and resources. IoT devices often operate across diverse communication bands and protocols, such as Bluetooth/Bluetooth Low Energy (BT/BLE), Wi-Fi, cellular networks (5G/6G), and Long Range (LoRa). These resource constraints further complicate the implementation of security measures. Devices that are powered by batteries face limitations that prevent the use of energy-intensive mechanisms such as AES-256 encryption or frequent firmware updates. Although low-power protocols like BLE and Long-Range Wide Area Network (LoRaWAN) help conserve energy, they inherently limit continuous security monitoring, necessitating trade-offs between robustness and device longevity. Additionally, bandwidth limitations in protocols such as Narrowband Internet of Things (NB-IoT) or Zigbee hinder real-time encrypted communication and prompt security updates, affecting monitoring and incident response capabilities. The balance between local and remote data processing presents further challenges [47].
- Regulatory compliance—ensuring that ZTA implementations adhere to relevant standards and data protection regulations—varying significantly across industries and regions—is crucial, especially given the global connectivity of these devices. Notable examples include the NIST 800-183 (Network of Things) publication [48], providing a comprehensive framework for defining and securing IoT systems with an emphasis on trustworthiness, data integrity, and secure communication. The Securing the Internet of Things guidance focuses on enhancing IoT security for critical infrastructure, with recommendations such as device segmentation, robust identity management, and continuous vulnerability assessments. Globally, the European Union Agency for Cybersecurity (ENISA) has delineated guidelines highlighting best practices aimed at enhancing IoT security in sectors like healthcare, smart cities, and industrial systems. It advocates for the integration of security-by-design principles, which integrate encryption, identity management, and data protection from the onset. Further, NIST SP 800-213A [49] specifies and assesses cybersecurity requirements throughout the procurement, deployment, and operation of IoT devices. Additionally, data privacy regulations like the Protection of Personal Information Act (POPIA) or General Data Protection Regulation (GDPR) are essential to safeguarding data. Collectively, these provide a comprehensive roadmap to foster compliance, mitigate vulnerabilities, and build resilient IoT ecosystems across industries.

As security remains a paramount concern for IoT devices, finding a workaround for these challenges is crucial to ensure ZTA is effectively implemented in IoT environments. Understanding these challenges and devising mitigation plans prior to implementation is vital. Another issue to consider is the use of SSI for NHIs, which has been extensively studied for human identities, and unlike for non-human identities, e.g., devices, applications, sessions, service accounts, etc. According to [50], although SSI applies to human-focused scenarios, the variety of devices, limited resources, and other restrictions pose significant issues for the broad SSI adoption in IoT ecosystems. Overcoming these issues is crucial to effectively integrating SSI into the wider IoT. Additionally, the solutions should investigate alternative methods for implementing SSI on highly resource-constrained devices, particularly in cases where IoT devices lack network connectivity.

## 5. ZTA Process and Use Case

This section discusses the process of implementing ZTA (in Section 5.1) as defined in [43] to set the scene. Section 5.2 builds ZTA principles alongside the processes and outlines a use case.

### 5.1. ZTA Process

**Figure 5:**
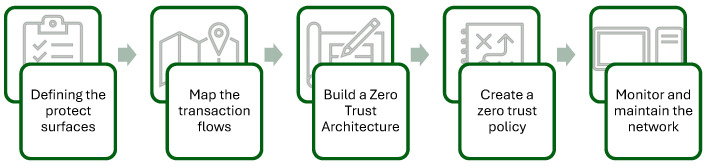
Five-Step process for Zero Trust implementation [47].

A study by [47] proposes a systematic five-step methodology for implementing ZTA within IoT environments. This structured approach ensures that ZTA security principles are consistently applied across the environment. These steps are illustrated in Figure 5.

- Defining the protected surfaces—fundamentally, the zero-trust model is predicated on the assumption that the network perimeter has been compromised. Consequently, it does not inherently trust entities within the environment, even if those entities have successfully authenticated at the perimeter [51]. In this step, the objective is to initially identify the most critical elements of Data, Applications, Assets, and Services (DAAS) that require protection (referred to as protected surfaces). This is crucial, as it enables organizations to determine the priority of security measures. Furthermore, it allows for the assessment of how any compromise of these elements impacts the value delivered, whether business or otherwise.
- Map the transaction flows—involves identifying the information flows among the DAAS elements. This includes flows within a protected surface, between protected surfaces, and with external elements, while assessing their current security maturity. This documentation specifies who or what requires access, when access is needed, and how it occurs to establish a baseline.
- Build a ZTA—this phase involves the conversion of insights derived from transaction flow mapping into concrete design elements, thereby establishing a foundation for ensuring comprehensive security in IoT ecosystems. It entails designing and implementing micro-segmentation and controls around the protected surface, segmenting the network to contain threats, and enforcing the principle of least privilege. Additionally, it also involves defining and locating PDP, PEP, and PA, as well as selecting tools and technologies that align with the organization’s objectives.
- Create a zero-trust policy—this step establishes detailed access rules that regulate resource access, based on factors such as identity, device health, location, etc., in adherence to the “never trust, always verify” security principle.
- Monitor and maintain the network—the final step involves continuous monitoring of all activities, alongside the analysis of logs for detecting anomalies. It also requires regular review and update of policies, as the Zero Trust framework is characterized as an ongoing, iterative process. This approach is essential to ensure compliance, enforce policies, identify vulnerabilities, and establish a feedback mechanism for optimal monitoring.

After comprehending the foundational principles of ZTA and examining the systematic approach necessary for its effective implementation within an IoT environment, we can move on to explore a practical application of these concepts. The subsequent section provides a high-level use case based on a real-world scenario. This example is designed to demonstrate how the integration of ZTA principles can effectively enhance security measures in IoT deployments. Through this illustrative scenario, readers will be able to visualize the practical benefits of ZTA, gaining insights into how this strategy can be tailored to secure non-human identities for IoT devices.

### 5.2. Use Case

**Figure 6:**
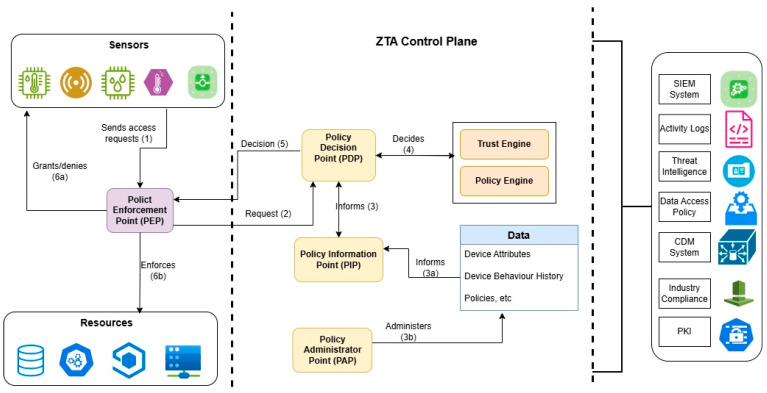
ZTA implementation.

The use case presents a practical ZTA example in the context of IoT devices in the field of smart agriculture. Figure 6 presents the illustration of the ZTA use case implementation. It illustrates how a variety of sensors initiate a request to access resources through the PEP, which both facilitates and enforces the decisions made by the PDP. The PDP employs a variety of metrics, including policies, activity logs, device behavior, etc., to ascertain the authorization permissions or entitlements of the requesting IoT device.

Within the context of smart agriculture, a network of low-power soil moisture sensors is deployed extensively across a farmland. These sensors, which are battery-operated and possess limited processing power, utilize low-bandwidth networks and lightweight protocols like Message Queuing Telemetry Transport (MQTT) for communication. To secure data, each sensor is assigned a unique identity through decentralized identifiers (DIDs). DIDs offer a lightweight, decentralized, and Self-Sovereign Identity (SSI) management solution that eliminates the need for centralized authorities. Throughout the authentication process, these DIDs maintain low memory and processing demands.

For the purpose of access control, the ZTA is implemented by enforcing policies at the edge gateway. This functions as a local trust anchor. Given the computational limitations of the sensors, the gateway conducts the bulk of the verification within a ZTA context. By bringing the policy decision point closer to the edge, rather than relying on a centralized cloud, this edge-based processing reduces the need to transport and process large datasets, thus minimizing latency. The gateway operates as a PEP, utilizing Role-Based Access Control (RBAC) to determine the sensors that are permitted to send data to external systems, such as irrigation controllers or cloud analytics platforms. This localized enforcement mitigates communication overhead and latency. Furthermore, sensors periodically relay data to the gateway, which monitors for anomalies like unexpected transmission patterns or firmware modifications. The ZTA principles [13] adapted in this use case include the following:

- Never trust, always verify—each sensor, gateway, controller, and cloud application must undergo authentication and authorization with every request.
- Assume breach—networks are configured and micro-segmented as if they are already compromised to restrict the blast radius and minimize the impact should a breach occur.
- Least privilege—RBAC or Attribute-Based Access Control (ABAC) is used at the edge gateway to restrict grants to the bare minimum privileges.
- Continuous monitoring—the entire solution is constantly monitored for anomalies, and this is implemented at the gateway.

This approach illustrates how ZTA principles, tailored for resource-constrained environments, can uphold robust security whilst accommodating scalability, interoperability, and operational efficiency in practical IoT applications.

## 6. Discussion

In the current technological landscape, traditional perimeter-based security approaches have demonstrated ineffectiveness, especially within IoT environments. Nevertheless, these have been effective in safeguarding human identities, with substantial advancements achieved in this area. However, the security of non-human identities has not kept pace, leaving them vulnerable. As NHIs grow rapidly, there is increasing pressure on industries to secure environments and demonstrate their commitment to securing products, especially with rising compromises in Information Technology (IT), operational technology, IoT, and smart home devices. These breaches involve more than just data exfiltration; they also include unauthorized access to misconfigured devices and software vulnerabilities, leading to insecure authentication and authorization. Organizations can address the dual pressure of demonstrating security progress and managing unauthorized access through ZTA. ZTA’s principles, like “never trust, always verify,” least privilege access, micro-segmentation, and continuous monitoring, provide a strong framework for mitigating risks such as compromised devices and lateral movement attacks.

ZTA has gained significant traction, especially in IoT environments and NHIs. However, the unique characteristics of NHIs must be considered during implementation. Existing studies acknowledge the security benefits of ZTA but often overlook these challenges. Key issues include the heterogeneity of IoT devices and their resource constraints, which may hinder computationally intensive security measures. The nature of ZTA requires continuous verification, which could impact performance in such devices. Although research has explored applying ZTA in IoT environments, there remains a gap in fully addressing implementation challenges. This study emphasizes the critical role of ZTA in addressing security challenges within IoT ecosystems, particularly in managing NHIs. Conventional perimeter-based security models are inadequate for dynamic IoT environments. A key insight is the importance of IAM in operationalizing ZTA for IoT. Unlike human identities, NHIs such as sensors and autonomous agents lack behavioral cues, complicating authentication and lifecycle management.

Integrating decentralized identity paradigms, such as SSI and DLTs, shows promise for enhancing trust and transparency, though adoption faces challenges from resource limitations and interoperability issues. The study also highlights the trade-offs of ZTA implementation. While ZTA improves security by reducing breaches and enabling granular access control, it introduces challenges like latency, scalability, and administrative overhead. Continuous verification and micro-segmentation, although effective, can impact real-time IoT operations, especially in resource-constrained environments. Lightweight techniques, edge-based policy enforcement, and adaptive trust evaluation mechanisms are critical for balancing security and performance. Furthermore, regulatory compliance and privacy considerations add complexity to ZTA deployment. The global nature of IoT ecosystems demands adherence to diverse data protection regulations, reinforcing privacy-by-design principles in identity frameworks. It is important to focus on developing standardized, interoperable solutions that integrate ZTA with emerging identity technologies, considering constraints like low processing power and energy limitations.

Based on the extensive literature reviewed, it becomes evident that the integration of ZTA with various emerging technologies holds significant potential to enhance the security of NHIs. Such integration is crucial, as it enables IoT systems to leverage the unique benefits and features offered by these advanced technologies, creating a more robust and secure environment for handling IoT data. The process of incorporating ZTA into these systems is instrumental in ensuring that all access requests are continuously verified and authenticated, thus minimizing potential security threats. This approach not only strengthens the overall security framework but also aligns with modern best practices. The relevant technologies that can be integrated with ZTA to achieve these enhancements include the following:

- DLTs—enhance security and transparency by ensuring secure transactions and data integrity while preventing unauthorized access. Their immutable nature is valuable for compliance and auditing, bolstering trust in decentralized networks, which is crucial in multi-party environments like supply chains.
- Edge computing—offers several advantages, including enhanced privacy, reduced latency, strengthened data security, and the capacity for real-time data analytics. Additionally, it remains reliable without constant network connectivity and provides rapid response times.
- AI and ML—enhance proactive threat detection with improved accuracy by learning and adapting to threats. They allow automated, risk-based access decisions, adjusting policies in real time instead of using static rules. AI also boosts efficiency by automating policy enforcement and threat responses, reducing manual work and human error.

In summary, this work positions ZTA as a practical necessity for securing IoT environments. Its successful implementation relies on balancing security rigor with operational efficiency, leveraging identity management innovations, and fostering collaboration across industries and regulatory bodies. As IoT adoption accelerates, advancing ZTA-based IAM solutions will be crucial for safeguarding the integrity and resilience of connected systems.

## 7. Conclusions

The rapid increase in the proliferation of IoT devices has introduced a significant paradigm shift in the realm of digital connectivity. This enables unprecedented levels of automation, seamless data exchange, and real-time decision-making capabilities across various sectors. However, this digital transformation has not come without its challenges, particularly regarding security. The expansion of IoT ecosystems has exposed critical vulnerabilities, especially in managing non-human identities within highly distributed and heterogeneous IoT environments. As illustrated in this study, traditional perimeter-based security models fall short in addressing these emerging challenges, emphasizing the need for innovative and adaptive security frameworks.

This paper introduces ZTA as a compelling alternative to conventional security measures, highlighting its foundational principles of continuous verification and least-privilege access, among others. By integrating ZTA into an IAM solution, a robust approach is developed for securing IoT ecosystems. Securing NHIs—for devices, sensors, service accounts, and autonomous agents—has transitioned from being optional to essential. ZTA provides a practical and structured framework for achieving this crucial security objective. While challenges in implementation persist, particularly in dealing with legacy systems and ensuring interoperability, the findings underscore the pivotal role of ZTA in advancing IoT device security. Embracing ZTA represents a necessary evolution towards securing the next generation of connected systems, fortifying them against emerging threats.

While this study has outlined the potential of ZTA based on robust IAM for securing NHIs within IoT ecosystems, numerous avenues remain open for future research. For instance, the field could benefit from the development of lightweight solutions specifically tailored for resource-constrained IoT devices. Additional research is required to tackle issues related to interoperability and standardization. These are crucial for the practical and scalable deployment of ZTA in securing the rapidly expanding IoT landscape. Such advancements will play a key role in ensuring that IoT systems are not only secure but also resilient against evolving cyberthreats, thereby enhancing their reliability and trustworthiness in a tech-savvy world.

## Figures and Tables

**Table 1:** Basic tenets and core components description [11,13,14,15].

Tenets/Components	Responsibility
Verify Explicitly	Emphasizes constant verification of all access requests, ensuring no entity is trusted by default.
Assume Breach	Assumes all access requests are potentially malicious, minimizing the blast radius for breaches.
Least Privilege Access	Provides necessary access to limit attack surface and breach impact.
Continuous Monitoring and Validation	Dynamically enforces security policies in real-time, ensuring continuous, valid, and secure access.
Enterprise Public Key Infrastructure (PKI)	Issues and logs certificates for various entities.
IAM System	Manages user accounts and identities, utilizing other systems for associated artifacts.
Security Information and Event Management (SIEM)	Obtains security data for review, aiding in policy adjustments and threat alerts.
Continuous Diagnostics and Mitigation (CDM)	Continuously assesses and responds to threats, informing the policy engine about asset access requests.
Micro-segmentation	Protects assets by segmenting the network to limit attackers’ lateral movement.
Threat Intelligence	Provides real-time data on vulnerabilities, malware, and attacks, facilitating the policy engine in denying access to compromised assets.
Activity Logs	Aggregates log data and network traffic to provide real-time insights into the system’s security status.
Data-Access Policies	Establishes a framework for resource access regulation predicated on user roles and requirements, thereby serving as the foundation for the allocation of permissions.
Industry Compliance	Guarantees adherence to regulatory compliance by implementing policy rules that are consistent with established security standards and frameworks.
Policy Enforcement Point (PEP)	Manages subject-resource connections.
Policy Administrator (PA)	Manages initiation and termination of communication links, contingent on the PE’s approval or denial.
Policy Engine (PE)	Grants, denies, or revokes resource access whilst logging these decisions.

**Table 2:** Comparing traditional security and ZTA models.

Category	Traditional Security Models	ZTA
Approach	Relies on perimeter-based security with trusted internal entities.	Assumes zero implicit trust, enforcing strict identity verification for all access.
	Uses firewalls, VPNs, and DMZs to protect network edges.	Employs continuous monitoring, micro-segmentation, and dynamic policy enforcement.
Key Gains	Simplicity: Easier to manage with clear network boundaries.	Improved Security: Up to 50% reduction in breach likelihood due to rigorous authentication and monitoring.
	Cost-effective: Lower initial investment in complexity and integration.	Scalability: Up to 30% faster integration with cloud services.
		Effective least privilege access reduces access risks.
Costs/Limitations	Insider Threats: Vulnerable to lateral movement by insiders or attackers. Scalability Issues: Challenging with expanding networks. Limited Visibility: Lacks continuous monitoring and detailed access control.	Complex Implementation: 25–30% increase in upfront investment for infrastructure overhaul and training. Administrative Overhead: 10–20% higher operational costs for monitoring and response. Performance Impact: Possible latency from frequent identity verification, though increasingly mitigated.

## Data Availability

Data are contained within the article.

## Abbreviations

The following abbreviations are used in this manuscript:

5G/6G	Cellular networks
ABAC	Attribute-Based Access Control
AES	Advanced Encryption Standard
AI	Artificial Intelligence
APIs	Application Programming Interface
ATOs	Account Take-overs
BT/BLE	Bluetooth/Bluetooth Low Energy
CDM	Continuous Diagnostics and Mitigation
DAAS	Data, Applications, Assets, and Services
DIDs	Decentralized IDentifiers
DLTs	Distributed Ledger Technologies
DMZs	Demilitarized Zones
DRL	Deep Reinforcement Learning
ENISA	European Union Agency for Cybersecurity
FIoT	Future IoT
FSM	Fuzzy Logic-Based State Management
GDPR	General Data Protection Regulation
IAM	Identity and Access Management
IDSs	Intrusion Detection Systems
IIoT	Industrial Internet of Things
IoT	Internet of Things
IP	Internet Protocol
IPSs	Intrusion Prevention Systems
IT	Information Technology
LoRa	Long Range
LoRaWAN	Long-Range Wide Area Network
MFA	Multi-Factor Authentication
ML	Machine Learning
MQTT	Message Queuing Telemetry Transport
NB-IoT	Narrowband Internet of Things
NHIs	Non-Human Identities
NIST	National Institute of Standards and Technology
PA	Policy Administrator
PE	Policy Engine
PEP	Policy Enforcement Point
PKI	Public Key Infrastructure
POPIA	Protection of Personal Information Act
PUFs	Physical Unclonable Functions
RBAC	Role-Based Access Control
SDN	Software-Defined Networking
SIEM	Security Information and Event Management
SSI	Self-Sovereign Identity
SSO	Single Sign-On
TPS	Transaction Per Second
VCs	Verifiable Credentials
VPNs	Virtual Private Networks
ZTA	Zero Trust Architecture
ZT-BlocIoT	Zero-Trust Blockchain-Enabled Framework
